# Can early weight loss, eating behaviors and socioeconomic factors predict successful weight loss at 12- and 24-months in adolescents with obesity and insulin resistance participating in a randomised controlled trial?

**DOI:** 10.1186/s12966-016-0367-9

**Published:** 2016-04-01

**Authors:** Megan L. Gow, Louise A. Baur, Mandy Ho, Kerryn Chisholm, Manny Noakes, Chris T. Cowell, Sarah P. Garnett

**Affiliations:** Institute of Endocrinology and Diabetes, The Children’s Hospital at Westmead, Locked Bag 4001, Westmead, NSW 2145 Australia; The Children’s Hospital at Westmead Clinical School, University of Sydney, Locked Bag 4001, Westmead, NSW 2145 Australia; Kids Research Institute, The Children’s Hospital at Westmead, Locked Bag 4001, Westmead, NSW 2145 Australia; Nutrition and Dietetics and Weight Management Services, The Children’s Hospital at Westmead, Locked Bag 4001, Westmead, NSW 2145 Australia; CSIRO Food and Nutritional Sciences, PO Box 10041, Adelaide BC, SA 5000 Australia

**Keywords:** Pediatric, Obesity, Insulin resistance, Predictors, Eating behaviors, Socioeconomic, Weight loss, RESIST

## Abstract

**Background:**

Lifestyle interventions in adolescents with obesity can result in weight loss following active intervention but individual responses vary widely. This study aimed to identify predictors of weight loss at 12- and 24-months in adolescents with obesity and clinical features of insulin resistance.

**Methods:**

Adolescents (*n* = 111, 66 girls, aged 10–17 years) were participants in a randomised controlled trial, the RESIST study, examining the effects of two diets differing in macronutrient content on insulin sensitivity. Eighty-five completed the 12-month program and 24-month follow-up data were available for 42 adolescents. Change in weight was determined by BMI expressed as a percentage of the 95th percentile (BMI95). The study physician collected socioeconomic data at baseline. Physical activity and screen time, and psychological dimensions of eating behavior were self-reported using the validated CLASS and EPI-C questionnaires, respectively. Stepwise multiple regressions were conducted to identify models that best predicted change in BMI95 at 12- and 24-months.

**Results:**

Mean BMI95 was reduced at 12-months compared with baseline (mean difference [MD] ± SE: -6.9 ± 1.0, *P* < 0.001) but adolescents had significant re-gain from 12- to 24-months (MD ± SE: 3.7 ± 1.5, *P* = 0.017). Participants who achieved greater 12-month weight loss had: greater 3-month weight loss, a father with a higher education, lower baseline external eating and parental pressure to eat scores and two parents living at home. Participants who achieved greater 24-month weight loss had: greater 12-month weight loss and a lower baseline emotional eating score.

**Conclusions:**

Early weight loss is consistently identified as a strong predictor of long-term weight loss. This could be because early weight loss identifies those more motivated and engaged individuals. Patients who have baseline factors predictive of long-term weight loss failure may benefit from additional support during the intervention. Additionally, if a patient does not achieve early weight loss, further support or transition to an alternate intervention where they may have increased success may be considered.

**Trial registration:**

Australian New Zealand Clinical Trial Registration Number (ACTRN) 12608000416392 https://www.anzctr.org.au/Trial/Registration/TrialReview.aspx?id=83071

## Background

Adolescent obesity is a well-recognised global public health concern, and effective treatment of affected individuals is a priority. Lifestyle interventions typically result in weight loss immediately following active treatment and after a short maintenance period (i.e. 6- to 12-months from baseline) but individual responses vary widely [1, 2]. Identification of predictors, at baseline or early in the intervention, associated with successful long-term weight loss may assist in tailoring treatment to the individual, identifying treatment non-responders and providing additional support as required.

There is a paucity of studies looking at predictors of obesity treatment success in adolescent only cohorts. However, several ‘mixed’ child and adolescent studies have identified early weight loss as an important predictor of later weight loss [3–6]. Early weight loss in these studies was measured between 4-weeks and 10-months into the intervention and predicted weight loss at follow-up between 16-weeks and 3-years from baseline. Other predictors of later weight loss identified in adolescents include: higher baseline body mass index (BMI) [3], greater baseline physical activity levels [4, 7] and male sex [6, 8]. There is an inconsistency in age as a predictor of treatment outcome, with both older [3] and younger age [5, 8, 9] being associated with successful weight loss.

The relationship between socioeconomic status (SES) and obesity treatment outcome is not clear. It has been assumed that a lower SES may negatively influence a parent’s ability to facilitate healthy eating patterns in their child [10]. One study found family adversity characteristics, including low parent education and history of a broken home, predictive of failed weight loss long-term [11]. In contrast, several studies have found that the family SES, including family structure and parental employment status, does not play a role in predicting obesity treatment outcome [3, 7, 8, 12, 13]. However, a study of longitudinal data published in 2015 from the US, indicated that gains in family income lead to significant decreases in BMI z scores in girls aged 2–6 years, suggesting a particular effect of SES status in younger girls compared with older girls and boys [14]. Additionally, low SES is more prevalent in certain ethnic groups, a factor which is thought to play a major role in the racial differences in observed obesity rates [15, 16].

Dysfunctional eating behaviors are known to play a role in the development of obesity in adolescents [17], but research into whether baseline eating behaviors can predict obesity treatment outcome is scarce. Only three studies have examined this, with no association being found between external or emotional eating and obesity treatment outcome [13, 18, 19]. Restrained eating at baseline was associated with greater weight loss at 10-months in one study only [18]. None of these studies measured eating behavior during or at the end of the intervention.

The aim of this study was to determine predictors of weight loss at 12-months (end of intervention) and 24-months (follow-up) in adolescents with obesity who participated in a weight loss trial. In particular we were interested in examining early weight change, eating behaviors and SES variables as predictors of weight loss.

## Methods

### Study participants and design

Adolescents in this study were participants in a randomised controlled trial (RCT), known as the Researching Effective Strategies to Improve Insulin Sensitivity in Children and Teenagers (RESIST) study. The study was an assessor blinded RCT consisting of a 12-month intervention and a 24-month follow-up, conducted at The Children’s Hospital at Westmead, Sydney, Australia. The study was approved by The Children’s Hospital at Westmead Human Research Ethics Committee (07/CHW/12) and Sydney South West Area Health, Western Zone (08/LPOOL/195). Written informed consent from parents and assent from the young people was sought prior to their enrolment in the study. The trial was registered with the Australian New Zealand Clinical Trial Register (ACTRN12608000416392). The methods have been described in detail elsewhere [20].

In brief, participants were recruited between February 2009 and December 2011. 24-month follow-up assessments were complete by December 2013. Eligible adolescents were: overweight or obese (International Obesity Task Force age-sex adjusted definitions [21]), aged 10 to 17 years, and with pre-diabetes or insulin resistance and at least one other clinical feature of insulin resistance including polycystic ovarian syndrome, hypertension, dyslipidaemia, non-alcoholic fatty liver disease and acanthosis nigricans [20]. Pre-diabetes was defined by the American Diabetes Association classification (impaired fasting glucose > 5.6 mmol∙L-1 and/or impaired glucose tolerance > 7.8 mmol∙L-1) [22], and insulin resistance was defined as a fasting insulin (pmol∙L-1) to glucose (mmol∙L-1) ratio greater than 20. Adolescents were ineligible if they had: type 1 or type 2 diabetes, contraindications to metformin therapy, secondary causes of obesity, been taking weight loss medication or medications known to cause weight gain, psychiatric disturbances, significant mental illness, an inability to take part in physical activity, weight more than 120 kg, and/or no parent who spoke English. Eligible adolescents were randomly assigned, stratified by weight, pubertal status and sex, using computer-based minimisation [23], to one of two dietary interventions and commenced on metformin therapy.

### Interventions

Details of the RESIST study intervention have been previously described in the protocol [20]. In brief, there were two dietary intervention arms: an increased protein diet group (40–45 % of total energy as carbohydrate, 30 % fat and 25–30 % protein), and a high carbohydrate diet group (55–60 % of total energy as carbohydrate, 30 % fat and 15 % protein). Other than the macronutrient content of the diet, the 12-month intervention was identical for both groups and incorporated 3 phases:

- 0 to 3-months: an intensive structured dietary intervention delivered by study dietitians (face-to-face visits at 0, 2, 6, 12 weeks with parents present and support at 4, 9 weeks)
- 4- to 6-months: an intensive exercise program (two 45-min sessions per week) delivered by personal trainers (at a gym or park) plus ongoing dietary support (SMS/email/phone) from study dietitians
- 7- to 12-months: a maintenance phase with ongoing dietary support (SMS/email/phone) from study dietitians

At completion of the 12-month intervention, adolescents were provided with standard healthy lifestyle advice by the study dietitian irrespective of randomisation group. Adolescents were given information about healthy snack options, appropriate portion sizes, label reading and physical activity recommendations. They were also instructed to continue with their metformin regimen until they were reviewed by their endocrinologist, which was scheduled 1- to 3-months following completion of the intervention. Following the 12-month visit participants received no contact, support or intervention from study dietitians until participants were contacted to organise attendance at the 24-month follow up.

### Measurements

Weight (kg) and height (cm) were measured by study nurses at baseline, 3-, 6-, 12- and 24-months using standard procedures. BMI (kg∙m^-2^), expressed as a percentage of the 95^th^ percentile (BMI95) for each particular child, was calculated from age and sex specific reference values [24]. BMI95 is used to describe changes in weight status due to its flexibility in describing heavier children (above the 97^th^ percentile) compared with BMI z score [24–26]. Change in BMI z score was considered to be inappropriate as more than 96 % of RESIST adolescents had a BMI greater than the 97^th^ percentile at baseline. Pubertal status was categorised according to the Tanner scale during a physician assessment at baseline, 12- and 24-months [27]. Demographic and socioeconomic information including number of parents living in the household, family income and mother and father education was collected by the study physician at baseline.

Psychological dimensions of eating behaviors were determined by the self-reporting Eating Pattern Inventory for Children (EPI-C) at baseline, 3-, 6-, 12- and 24-months which has been validated in a paediatric population [28]. The 20 item questionnaire consisted of 4 subscales: external eating (eating in response to external food cues), emotional eating (eating in response to negative emotions), dietary restraint (cognitive determination and efforts to restrict food intake in order to control body weight) and parental pressure to eat. Responses to each item were listed in a 4-point Likert scale format (1 = not at all, 2 = sometimes, 3 = mostly, 4 = always). Scores for each subscale were obtained by dividing the total scores by the total number of items in the respective subscale, with each subscale having a score ranging from 1 to 4. Higher scores in the respective subscales are indicative of greater external eating, emotional eating, dietary restraint or parental pressure to eat.

Typical time per week spent in a range of leisure time activities was self-reported by adolescents using the Children’s Leisure Activities Study Survey (CLASS) questionnaire at baseline, 3-, 6-, 12- and 24-months; a reliable and valid measure in a pediatric population [29]. Leisure time activities included physical activities and sedentary behaviors, such as recreational screen time activities. From the CLASS questionnaire, total time spent in daily physical activity (PA) and recreational screen time (ST) was calculated [29].

### Statistical analysis

Data were assessed for normality and analysed using IBM ® SPSS Statistics Software for Windows, version 22 (IBM Corp, Armonk, NY, USA). Baseline differences between 24-month attendees and non-attendees were determined using independent sample t tests for continuous data and chi-squared tests for categorical data. Consistent with an intention-to-treat approach, changes in BMI95, eating behaviors and leisure time activities over time were examined using linear mixed models with an unstructured covariance framework and Least Significant Difference adjusted post-hoc tests for participants with a baseline measure. In linear mixed models, maximum likelihood estimates provide approximations for missing data. Non-parametric data were log or square root transformed as appropriate. Sex and pubertal status were tested in the model but were not significant, hence results have been reported as unadjusted models and expressed as estimated marginal means (EMM) with standard error (SE) of the mean for normal data or geometric marginal means (GMM) with 95 % confidence intervals (CI) for non-parametric data. Transformation of emotional eating scores did not normalise the data, hence changes over time were analysed using Wilcoxon matched-pair signed-rank test and reported as median and range.

Change in weight status was measured by change in BMI95. A reduction in BMI95 is described as ‘weight loss’ and a gain in BMI95 is described as ‘weight gain’. Change in BMI95 at 3-months was categorised into 4 groups: gained weight (gain in BMI95), lost 0 to 4.99 % of BMI95, lost 5 to 9.99 % of BMI95, lost ≥ 10 % of BMI95. Analysis examining differences between those who gained weight and those who lost at least 5 % of BMI95 was conducted using independent sample t test for continuous data and chi-squared tests for categorical data. Odds ratios (OR) were used to examine the magnitude of associations.

Correlations between changes in BMI95 at 3-, 12- and 24-months and other variables were assessed by Pearson’s correlation coefficient for normally distributed variables, Spearman’s correlation coefficient for non-parametric variables and Kendall’s tau correlation coefficient for categorical variables. Variables explored included age, pubertal stage, sex (male = 1, female = 2), baseline BMI95, SES factors, psychological dimensions of eating behavior and leisure time activities. Coding for socioeconomic factors, number of parent living in the household (single-parent = 1, two-parent = 2), parent education (<year 12 = 1, completed year 12 = 2, completed tertiary/trade degree = 3) and family income (<AU$31,200/year = 1, AU$31,200-AU$67,599/year = 2, AU$67,600–$103,999/year = 3, ≥AU$104,000/year = 4) was completed to allow exploration of associations. Stepwise multiple regression was conducted with significantly associated variables to identify models that best predicted change in BMI95 at 3-, 12- and 24-months. Significant (*P* < 0.05) variables or those considered to be theoretically relevant were included in the predictive models. One outlier (defined as ≥ 3 SDs from the regression line for predicting BMI95 at 3-, 12- and 24-months) was identified but included in the analyses as it is known to be a true result and did not impact significantly on the models. The assumptions of modelling were tested and met.

## Results

Baseline characteristics of the 111 adolescents (59 % girls) recruited to the RESIST study are described in Table 1. Eighty five adolescents (56 % girls) completed the 12-month RESIST intervention and 42 (60 % girls) returned for follow-up at 24-months. At baseline, the 42 adolescents who attended the 24-month follow-up were younger (mean difference [MD] ± SE: 0.86 ± 0.36 years, *P* = 0.018) and more likely to be pre-pubertal (50 % versus 19 %, *P* = 0.012) compared with non-attenders but BMI95, eating behaviors and time spent in PA and ST did not differ between groups. Previous analyses of insulin sensitivity, weight, body composition, eating behaviors and leisure time activities found no differences between diet intervention groups at any time point [30–33]. Therefore diet groups were combined for this analysis and report.

**Table 1:** Baseline characteristics of study cohort, *n* = 111

Age and sex		
Age, years, median [range]		13.2 [10.1–17.4]
Girls		66 (60)
Pubertal status (*n* = 110)		
Tanner stage	1	14 (13)
	2	20 (18)
	3	24 (22)
	4	31 (28)
	5	21 (19)
Anthropometry		
BMI z score, mean ± SD		2.36 ± 0.29
BMI95, mean ± SD		132.3 ± 21.1
Obese		107 (96)
Eating behaviors^a^ (*n* = 109)		
External eating score, GMM [95%CI]		1.7 [1.6–1.9]
Emotional eating score, median [range]		1.5 [1.0–3.0]
Dietary restraint score, mean ± SD		2.6 ± 0.6
Parental pressure to eat score, mean ± SD		2.0 ± 0.8
Leisure time activities		
Physical activity (*n* = 109), mins/day, GMM [95%CI]		82 [71–93]
Screen time, mins/day (*n* = 108), GMM [95%CI]		223 [198–248]
Socioeconomic factors		
Parents in household (*n* = 100)		
Single-parent household		27 (27)
Two-parent household		73 (73)
Mother’s education (*n* = 100)		
Below year 12		37 (37)
Completed year 12		14 (14)
Completed technical school or tertiary study		49 (49)
Father’s education (*n* = 88)		
Below year 12		37 (42)
Completed year 12		11 (13)
Completed technical school or tertiary study		40 (45)
Family income (*n* = 97)		
< AU$31,200/year		29 (30)
AU$31,200–$67, 599/year		38 (39)
AU$67,600–103,999/year		17 (18)
≥ AU$104,000		13 (13)

Unless otherwise stated, *n* = 111, expressed as n (%)

*Abbreviations*: *AU* Australian Dollar, *BMI* body mass index, *BMI95* BMI expressed as a percentage of the 95^th^percentile, *CI* confidence interval, *GMM* generalised marginal mean, *SD* standard deviation

^a^Scores for each subscale range from 1 to 4; higher scores indicate greater external eating, emotional eating, dietary restraint or parental pressure to eat

### Effects of intervention and follow-up on weight, eating behaviors and leisure activities

**Fig. 1:**
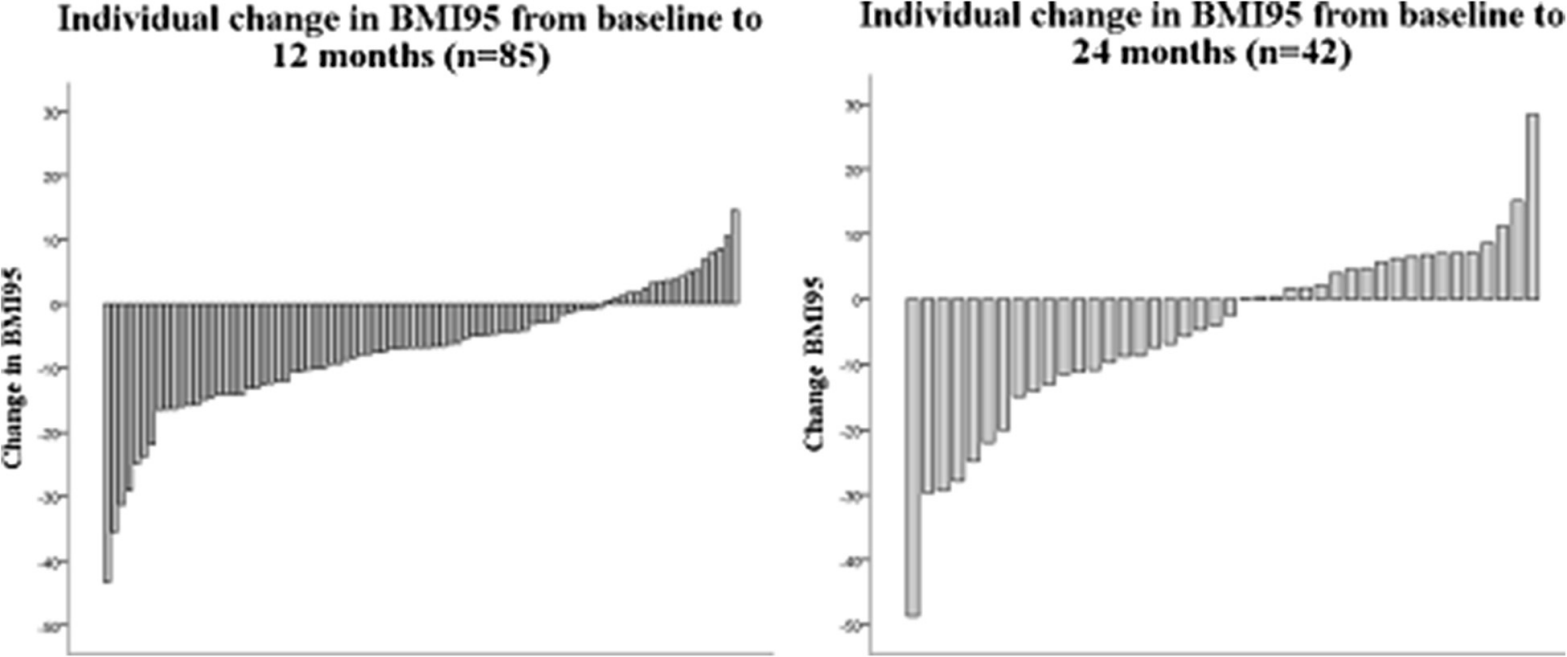
Individual changes in BMI95 at 12 and 24 months

Figure 1 shows individual changes in BMI95 at 12- and 24-months. At 12-months, 67 of the 85 adolescents (79 %) who completed the intervention had lost weight (mean ± SD: 10.6 ± 8.3 reduction in BMI95) while at 24-months, 23 of the 42 adolescents (55 %) who returned for follow-up had lost weight (mean ± SD: 14.6 ± 11.4 reduction in BMI95) compared with baseline. Intention-to-treat analysis of the 111 adolescents recruited to the trial indicated that BMI95 was reduced at 12-months compared with baseline (MD ± SE: -6.9 ± 1.0, *P* < 0.001). However, at 24-months BMI95 was not significantly different from baseline (MD ± SE: -3.1 ± 1.8, *P* = 0.082). From 12- to 24-months adolescents had significant gain in BMI95 (BMI95; MD ± SE: 3.7 ± 1.5, *P* = 0.017) as well as significant gain in actual body weight (MD ± SE: 8.8 ± 1.4 kg, *P* < 0.001; range: -9.4 to 35.0 kg). Weight gain during the 12-month intervention did not increase the likelihood that the adolescent would be a 24-month follow-up non-attender (OR 1.2 [95 % CI: 0.5 to 2.8], *P* = 0.635).

Both at 12- and 24-months, external eating scores were significantly lower than at baseline (MD ± SE: 0.37 ± 0.08, *P* < 0.001 and 0.38 ± 0.11, *P* = 0.001). Emotional eating was also significantly reduced compared with baseline at 24-months only with the median score dropping from 1.25 at baseline to 1.0 at 24-months (Z = -2.63, *P* = 0.008). Neither dietary restraint nor parental pressure to eat scores were significantly different from baseline at 12- or 24-months. At 12-months PA and ST levels were not different from baseline (GMM [95%CI]: 84 [72 – 96] and 194 [166 – 224] mins/day, respectively), nor were they different from baseline at 24-months (71 [50 – 97] and 219 [176 – 265] mins/day, respectively).

### Correlates of weight loss at 12- and 24-months

Associations between change in weight at 3-, 12- and 24-months and baseline age, puberty, sex, psychological dimensions of eating behavior, SES measures and leisure time activities are reported in Table 2. Weight loss represents a negative change in weight. Therefore negative correlations in Table 2 indicate that an increase in the variable of interest is associated with weight loss.

**Table 2:** Correlation coefficients of BMI95 at 3-, 12- and 24-months

	Change BMI95 baseline to 3 m	Change BMI95 baseline to 12 m	Change BMI95 baseline to 24 m
Change BMI95 baseline to 3mo^a^		0.451**	0.215
Change BMI95 baseline to 12mo^a^			0.706**
Sex^b^ *(coding: male = 1, female = 2)*	-0.068	-0.102	0.005
Age, years^a^	0.313**	-0.037	-0.056
Pubertal (stage 1 to 5)^b^	0.150*	-0.093	-0.029
BMI95^a^	-0.210*	0.116	0.161
External eating score^c^	-0.133	0.250*	0.304
Dietary restraint score^a^	-0.008	-0.059	-0.128
Parental pressure to eat score^a^	0.004	0.228*	0.106
Emotional eating score^c^	-0.092	0.118	0.336*
Baseline daily screen time^a^ (mins/day)	-0.116	-0.233*	0.046
Baseline daily physical activity^c^ (mins/day)	0.215*	0.331*	0.355*
Number of parents living in household^b^ *(coding: one = 1, two = 2)*	-0.022	0.050	0.006
Mother’s education^b^ *(coding: <year 12 = 1, completed year 12 = 2, completed tertiary/trade degree = 3)*	0.033	-0.126	-0.189
Father’s education^b^ *(coding as for ‘Mother’s education’)*	-0.12	-0.275**	-0.171
Family income^b^ *(coding: <$31,200/yr = 1, $31,200–$67,599/yr = 2, $67,600-$103,999/yr = 3, ≥$104,000/yr = 4)*	-0.043	-0.256**	-0.362**

All variables were measured at baseline unless otherwise specified. BMI95: body mass index expressed as a percentage of the 95^th^percentile. *p < 0.05; **p < 0.01

^a^Pearson’s correlation coefficient (r), ^b^Kendall’s τ (tau), ^c^Spearman’s ρ (rho)

Greater weight loss at 12-months was significantly associated with: lower external eating and less parental pressure to eat at baseline, higher family income, higher paternal education and greater weight loss at 3-months. Lower PA and greater ST at baseline were correlated with increased weight loss at 12-months. Greater weight loss at 24-months was significantly associated with: greater weight loss at 12-months, lower emotional eating score at baseline and a higher family income. Similar to the findings at 12-months, lower baseline PA was associated with greater 24-month weight loss.

We also examined associations between changes in eating behaviors from baseline to 3-, 12- and 24-months with changes in weight from baseline to 12- and 24-months. The only significant correlation was a weak association between an increase in parental pressure to eat from baseline to 12-months and greater weight loss at 12-months (*r* = -0.224, *P* = 0.049). No other significant correlations were observed.

### Predictive models for weight loss at 12- and 24-months

The best predictive model for weight loss at 12-months, explaining 53 % of the variation, is shown in Table 3. Change in weight in the first 3-months alone predicted 20 % of the variance in change in weight at 12-months. The model indicates that participants who had achieved more weight loss at 12-months had: lost more weight at 3-months, a father with a higher level of education, a lower baseline external eating score, two parents residing in the household and less pressure to eat from parents at baseline.

**Table 3:** Predictive model explaining the change in BMI95 at 12 months

Predictor	Regression coefficient	95 % CI	R^2^ change	*p*
Change BMI95 baseline to 3mo	1.123	0.77 to 1.48	0.246	<0.001
Fathers education	-3.826	-5.65 to -2.00	0.123	<0.001
Baseline external eating score	4.438	1.53 to 7.35	0.076	0.003
Parent marital status	5.583	0.79 to 10.38	0.048	0.015
Baseline parental pressure to eat score	2.647	0.36 to 4.93	0.038	0.024
Final adjusted R^2^			0.531	<0.001

Table 4 shows the best predictive model for change in weight at 24-months, explaining 55 % of the variation. The model indicates that participants who had greater weight loss at 12-months and lower baseline emotional eating scores had greater weight loss at 24-months. Early weight loss was not predictive of weight change at 24-months (*R*^2^ = 0.046; *P* = 0.171).

**Table 4:** Predictive model explaining the change in BMI95 at 24 months

Predictor	Regression coefficient	95 % CI	R^2^ change	*p*
Change BMI95 baseline to 12mo	0.917	0.64 to 1.25	0.513	<0.001
Baseline emotional eating score	5.257	-0.50 to 11.01	0.041	0.072
Final adjusted R^2^			0.554	<0.001

### Post hoc analysis – early weight loss

**Fig. 2:**
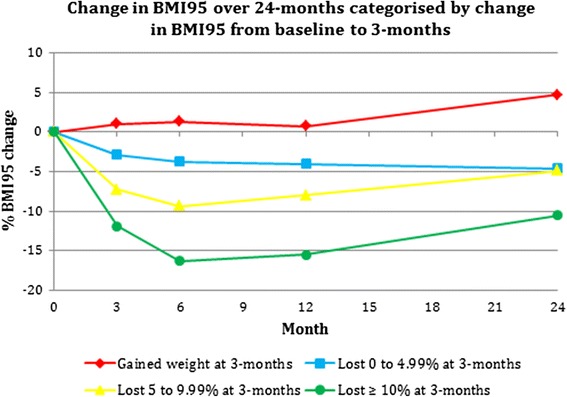
Percentage change in BMI95 at 6-, 12- and 24-months according to percentage change in BMI95 from baseline to 3-months

Early (3-month) weight loss was a strong predictor of weight loss at 12-months, which predicted weight loss at 24-months, hence post hoc analysis was conducted to further explore this relationship and to identify variables predictive of early weight loss. During the first 3-months of intervention, 90 adolescents lost weight (mean BMI95 change ± SD: -7.13 ± 4.08) and 16 gained weight (mean BMI95 change ± SD: 1.30 ± 1.30). Of those who lost weight, 45 had <5 % reduction, 38 had a 5 to 9.99 % reduction and 7 had ≥10 % reduction in BMI95 (Fig. 2).

#### Features of early weight change

Of the adolescents who lost ≥5 % BMI95 at 3-months, 62 % and 52 % maintained this at 12- and 24-months, respectively. Additionally, adolescents who gained weight during the first 3-months were less likely to have lost weight during the 12-month intervention (OR 3.2 [95 % CI: 1.5 to 7.0], *P* = 0.018), or at the 24-month follow-up (OR 2.8 [95 % CI 1.8 to 4.3], *P* = 0.005) and significantly more likely to have dropped out at 12-months (OR 2.8 [95 % CI: 1.3 to 5.9], *P* = 0.016).

#### Correlates of and predictive model for early weight loss

Correlation analysis indicated that younger, pre-pubertal participants with lower PA levels and higher BMI95 at baseline achieved greater weight loss at 3-months (Table 2). Reductions in both external eating and parental pressure to eat scores from baseline to 3-months was also associated with greater early weight loss (rho = 0.361, *P* < 0.001 and *r* = 0.198, *P* = 0.046, respectively). No other significant correlations were observed. Using multiple regression, the best predictive model accounted for 33 % of the variation (Table 5). The model indicated that younger participants with lower baseline PA levels, higher baseline BMI95 and reductions in external eating and parental pressure to eat from baseline to 3-months had greater weight loss at 3-months.

**Table 5:** Predictive model explaining the change in BMI95 at 3 months

Predictor	Regression coefficient	95 % CI	R^2^ change	*p*
Age, years	0.811	0.355 to 1.267	0.132	0.001
Change in external eating score baseline to 3mo	2.393	0.838 to 3.949	0.087	0.003
Baseline physical activity (mins/day)	0.019	0.005 to 0.033	0.039	0.006
Baseline BMI95	-0.051	-0.094 to -0.008	0.036	0.019
Change in parental pressure to eat score baseline to 3mo	1.606	0.075 to 3.137	0.031	0.040
Final adjusted R^2^			0.325	<0.001

## Discussion

Our results indicate that weight loss at 3-months was a strong predictor of weight loss at 12-months. Higher paternal education, living in a household where two parents reside, reporting to be less influenced to eat by external food cues and less pressure to eat from parents at baseline also predicted greater weight loss at 12-months. 24-month weight loss was best predicted by 12-month weight loss and adolescent-reported absence of eating in response to negative emotions at baseline.

Previous studies in children and adolescents have also found initial treatment success to be predictive of weight loss at follow-up [3–6]. Findings from two of these studies suggest it may be possible to identify treatment non-responders earlier than 3-months [5, 6]. Specifically, the study by Goldschmidt and colleagues identified weight loss at 8-weeks into a 20-week intervention to be the best predictor of weight loss at 20-weeks and 2-years following treatment [5]. Additionally, the Look AHEAD trial in adults with type 2 diabetes has identified weight loss at 1- and 2-months into treatment to be predictive of weight loss at 1-, 4- and 8-years [34, 35]. The consistent identification of early weight loss as a strong predictor of long-term weight loss in adult, adolescent and childhood literature challenges the necessity of RCTs in obesity treatment research. If early weight loss is not achieved the patient may benefit from transition to an alternate intervention rather than remaining in an intervention where they are unlikely to achieve long-term success. Alternatively, failure to achieve weight loss early in the intervention may indicate that the participant lacks other factors necessary for success, such as motivation or commitment to the intervention. In this case, lack of early weight loss may indicate that the patient requires additional support.

In our study, younger age was the most influential predictor of weight loss at 3-months. Other studies, including one in a real-life clinical setting [36], have also identified that younger participants do better in weight loss trials, indicating that weight loss interventions may be more successful if commenced at a younger age [5, 8, 9, 36]. As early weight loss was strongly predictive of 12-month weight loss, initiation of obesity treatment in younger adolescents is also likely to influence long-term weight loss. Furthermore, 24-month follow-up attendees in our study were younger than non-attendees. This provides further evidence to support the initiation of trials in younger adolescents as attendance at group based sessions as well as attending more clinic appointments have also been identified as important factors in achieving greater weight loss [6, 36].

To our knowledge, our previously published paper examining RESIST study data (up to 6-months) is the only paper to have reported on the impact of an obesity treatment intervention on psychological dimensions of eating behaviors, i.e. external, emotional and restrained eating, in free-living adolescents with obesity [31]. Overall, the implementation of structured dietary advice appears to have a positive impact on eating behaviors long-term with external and emotional eating being reduced at 24-months compared with baseline. Advocating regular eating through implementation of prescriptive dietary advice may therefore assist adolescents to alter the way they deal with external food cues and prevent eating in response to negative emotion. Additionally, this study is the first to show that baseline external and emotional eating styles can predict long-term weight loss in adolescents with obesity receiving obesity treatment. Patients who have high external or emotional eating styles at baseline may benefit from increased support during the intervention to increase the likelihood of success. We also found that short-term reductions in external eating and parental pressure to eat predicted 3-month weight loss. However, this did not extend beyond 3-months.

The role of restrained eating for weight loss during an obesity treatment program in adolescents is not clear. Previously published results from the RESIST study demonstrated that an increase in dietary restraint was associated with increased weight loss during the first 6-months of the study [31]. This is similar to what has been reported in adult studies [37–39]. However, in the present study we found no associations between restrained eating and weight loss in RESIST participants at 3-, 12- or 24-months. Similarly, other studies in adolescents have found no such relationship [13, 19]. Reported inconsistencies in the relationship between dietary restraint and obesity treatment outcomes is in line with the theory of restrained eating [40, 41]. This theory acknowledges that some dietary restraint is necessary for weight loss but that the cognitive effort required to eat less may become too difficult to maintain. The diet may then be abandoned altogether, resulting in a likelihood of overeating above non-dieting individuals. The difficulty in achieving the fine balance between self-control and loss of control may account for the discrepancies observed in studies.

Obesity treatment interventions typically result in weight loss in children and adolescents immediately following active treatment and after a short maintenance period, i.e. up to 12-months from baseline [1, 2]. However, obesity treatment is often followed by weight regain; therefore long-term weight loss maintenance is the main challenge [42, 43] and hence identifying predictors of successful weight loss maintenance is crucial. In our study, 12-month weight loss was highly predictive of 24-month weight loss but early weight loss was not. This may be because the sample size was not large enough to identify an association.

We also noted significant associations between lower PA and higher ST levels at baseline and greater weight loss. This is in contrast to a previous study in adolescents where higher baseline PA levels were associated with greater weight loss at 9 months [4]. Our findings suggest that adolescents with lower baseline PA and higher ST levels may have more to gain from a lifestyle intervention that incorporates a PA component. Consistent with one recent study, our study emphasises the importance of SES in achieving weight loss in adolescents [11]. Both father’s education and living in a two-parent household were significant predictors of 12-month weight loss and higher income was associated with increased weight loss at 12- and 24-months.

There are several factors which limit the application and generalizability of our findings. Firstly, all adolescent participants had clinical insulin resistance and/or pre-diabetes, and were on metformin therapy, and hence findings may not be applicable to the general population. Secondly, we were only able to retain 38 % of our original study cohort at 24-months. This may have affected our ability to identify some associations. High drop-out rates are a common problem in longer-term trials, ranging from 10 to 60 % at 12-months in child and adolescent obesity treatment interventions [2]. A higher drop-out rate in participants receiving structured dietary advice compared with general lifestyle advice has also been reported in young women, suggesting that the rigid diet structure may not be compatible with some young people’s lives [44]. We were not able to provide participants with ongoing support from 12- to 24-months. Contact with participants during this time may have assisted retention rates and/or maintenance of weight loss. However, findings from another outpatient lifestyle intervention known as the ‘Obeldicks’ study in 663 4-16 year olds demonstrated that weight loss during a 12-month intervention could be sustained at 5-years with similar drop-out rates as seen in our study [45]. We also acknowledge that there are many other important potential predictors of weight loss success (such as level of parental support, motivation of participant and mother’s psychopathology) that were not assessed. Finally, self-reporting of eating behaviors using the EPI-C, as in our study, is a known limitation.

## Conclusions

This study provides evidence that, even in adolescents with obesity and clinical insulin resistance, early weight loss is a strong predictor of long-term obesity treatment outcome. Patients who do not achieve early weight loss may benefit from transition to an alternate intervention. Additionally, identification of characteristics that predict long-term obesity treatment outcome, including early weight change and baseline eating behavior variables, may assist in tailoring treatment to the individual and allow provision of extra support to those predicted to require it.

## Abbreviations

BMI: body mass index
BMI95: BMI expressed as a % of the 95^th^percentile
CI: confidence interval
CLASS: children’s leisure activities study survey
EMM: estimated marginal mean
EPI-C: eating pattern inventory for children
GMM: geometric marginal mean
MD: mean difference
OR: odds ratio
PA: physical activity
RCT: randomised controlled trial
RESIST: researching effective strategies to improve insulin sensitivity in children and teenagers
SE: standard error
SES: socioeconomic status
ST: screen time
